# Cell Growth Inhibition, DNA Fragmentation and Apoptosis-Inducing Properties of Household-Processed Leaves and Seeds of Fenugreek (*Trigonella Foenum-Graecum* Linn.) against HepG2, HCT-116, and MCF-7 Cancerous Cell Lines

**DOI:** 10.3390/cimb45020060

**Published:** 2023-01-19

**Authors:** Shaimaa G. Abdel Salam, Mohamed M. Rashed, Nabih A. Ibrahim, Emam A. Abdel Rahim, Hadeil Muhanna Alsufiani, Rasha A. Mansouri, Mohamed Afifi, Ammar Al-Farga

**Affiliations:** 1 Food Technology Research Institute, Agricultural Research Center, Giza P.O. Box 12613, Egypt; 2 Biochemistry Department, Faculty of Agriculture, Cairo University, Giza P.O. Box 12613, Egypt; 3 Biochemistry Department, Faculty of Sciences, King Abdulaziz University, Jeddah 21959, Saudi Arabia; 4 Experimental Biochemistry Unit, King Fahd Medical Research Center, King Abdulaziz Unversity, Jeddah 21959, Saudi Arabia; 5 Department of Biochemistry, College of Sciences, University of Jeddah, Jeddah 21959, Saudi Arabia; 6 Department of Biochemistry, Faculty of Veterinary Medicine, Zagazig University, Zagazig P.O. Box 44519, Egypt; 7 Najla Bint Saud Al Saud Center for Distinguished Research in Biotechnology, Jeddah 21577, Saudi Arabia

**Keywords:** fenugreek, household processes, apoptosis, MCF-7, HepG2, HCT-116

## Abstract

Household processing of fenugreek seeds and leaves, including soaking, germination, and boiling of the seeds, and air-drying of the leaves, has improved the levels of human consumption of the bitter seeds and increased the shelf life of fresh leaves, respectively. The potential anticancer activity of either unprocessed or processed fenugreek seeds or leaves and the relative expression of pro-apoptotic and anti-apoptotic genes of the studied cancerous cell lines exposed to IC_50_ crude extracts was investigated to observe the apoptotic-inducing property of this plant as an anticancer agent. The protein expression of IKK-α and IKK-β, as inhibitors of NF-KB which exhibit a critical function in the regulation of genes involved in chronic inflammatory disorders, were studied in the tested cancerous cell lines. In this study, the anticancer activity of household-processed fenugreek leaves and seeds against HepG2, HCT-116, MCF-7, and VERO cell lines was measured using an MTT assay. DNA fragmentation of both HepG2 and MCF-7 was investigated by using gel electrophoresis. RT-PCR was used to evaluate the relative expression of each p53, caspase-3, Bax, and Bcl-2 genes, whereas ELISA assay determined the expression of caspase-3, TNF-α, and 8-OHDG genes. Western blotting analyzed the protein-expressing levels of IKK-α and IKK-β proteins in each studied cell line. Data showed that at 500 µg mL^−1^, ADFL had the highest cytotoxicity against the HepG2 and HCT-116 cell lines. Although, each UFS and GFS sample had a more inhibitory effect on MCF-7 cells than ADFL. Gel electrophoresis demonstrated that the IC_50_ of each ADFL, UFS, and GFS sample induced DNA fragmentation in HepG2 and MCF-7, contrary to untreated cell lines. Gene expression using RT-PCR showed that IC_50_ doses of each sample induced apoptosis through the up-regulation of the p53, caspase-3, and Bax genes and the down-regulation of the Bcl-2 gene in each studied cell line. The relative expression of TNF-α, 8-OHDG, and caspase-3 genes of each HepG2 and MCF-7 cell line using ELISA assays demonstrated that ADFL, UFS, and GFS samples reduced the expression of TNF-α and 8-OHDG genes but increased the expression of the caspase-3 gene. Protein-expressing levels of IKK-α and IKK-β proteins in each studied cell line, determined using Western blotting, indicated that household treatments decreased IKK-α expression compared to the UFS sample. Moreover, the ADFL and SFS samples had the most activity in the IKK-β expression levels. Among all studied samples, air-dried fenugreek leaves and unprocessed and germinated fenugreek seeds had the most anti-proliferative and apoptotic-inducing properties against human HepG2, MCF-7, and HCT-116 cell lines, as compared to the VERO cell line. So, these crude extracts can be used in the future for developing new effective natural drugs for the treatment of hepatocellular, breast, and colon carcinomas.

## 1. Introduction

Cancer remains an aggressive cause of death on a global scale despite significant efforts. The chemotherapeutic agents currently in use have negative side effects and have not performed as expected. Medicinal herbs have been used for ages to cure a variety of illnesses and are also known to have anticancer properties [1].

Tumor chemoprevention is a procedure used to decelerate, degenerate, or prevent the cross-mechanism of tumorigenesis, including blocking its important morphogenetic stages, such as preneoplasia, neoplasia, and metastasis. Alternatives such as naturally occurring phytochemicals found in food are increasingly preferred over manufactured medications for a number of reasons, including less (or no) toxicity or side effects, improved availability, and safety. Programmed cell death (PCD)-type I (apoptosis), PCD-type II (autophagic cell death), and necrosis are some of the mechanisms involved in cell death produced by plant products [2]. 

A complex biological process called apoptosis is crucial for removing damaged cells from living things. A type of controlled cell death known as apoptosis is characterized by distinctive morphological and biochemical alterations in the cells. The apoptotic cascade is fueled by a variety of proteins. They can be assessed utilizing common analytical procedures based on protein detection. Caspases are enzymes (i.e., proteases), and their activity can be used to determine the stage and rate of apoptosis, particularly when they are cleaving the peptide bonds of the right substrate. In humans, there are eleven isoenzymes of caspases, seven of which (caspases 2, 3, 6, 7, 8, and 10) are involved in apoptosis [3]. Apoptosis can occur through a number of different pathways, including nuclear condensation, DNA breakage, phosphatidyl serine externalization, membrane blabbing, and loss of mitochondrial membrane potential [4].

The I kappa B protein kinase complex, often known as IKK, is a 700 kDa protein that can phosphorylate the conserved N-terminal serine residues of IκB-α and IκB-β and is triggered by inflammatory signals (such as IL-1, TNF-α, bacterial LPS, viruses, and oxidants) [5]. The IKK complex is made up of a regulatory component called IKK-/NEMO/IKKAP1 and two catalytic subunits called IKK-α (or IKK-1) and IKK-β (IKK-2) [6]. IKK-α and IKK-β are Ser/Thr kinases with similar structures that may form homodimers and heterodimers in vitro and can directly phosphorylate IκB-α and β at the appropriate locations when in the purified recombinant form [7]. The activity of both IKK-α and IKK-β was demonstrated to be dependent on phosphorylation at specific serine residues (Ser-176/180 of IKK-α and Ser-177/181 of IKK-β) found in the T (activation) loop of the kinase domain. By substituting glutamate for these residues, which mimics the effect of phosphoserine, IKK-α and IKK-β’s activity was generated constitutively [8]. Studies concerning animals deficient in each IKK subunit revealed that IKK-α plays a role during the embryonic development of the skin and skeletal system, while IKK-β is essential for the activation of IKK in response to TNF and other pro-inflammatory stimuli; therefore, despite the similar structural similarities between IKK-α and IKK-β, this suggests their shared functional role in NF-κB activation [6].

Fenugreek, or *Trigonella foenum-graecum*, is a traditional herbal remedy used for the treatment of diabetes, high cholesterol, wounds, inflammation, digestive problems, etc. It is also thought to have anti-tumor characteristics; however, the exact mechanisms for this activity are still unclear [9]. Numerous studies on fenugreek have demonstrated that it contains active chemical components that are beneficial, such as steroidal sapogenins, dietary fiber, galactomannans, antioxidants, and amino acids such as 4-hydroxyisoleucine, that have the potential to treat hypocholesterolemia and hypoglycemia and have anti-diabetic, anti-leukemic, antipyretic, anti-nociceptive, anti-fertility activity, and anti-tumor properties. Fenugreek leaves or seeds and their biological components have an important role in tumor inhibition through modulating the activity of numerous genes, including the induction of apoptosis and tumor suppressor genes and also the inhibition of tumor necrosis factors [10].

The aim of this work is to evaluate the anti-proliferative activity of air-dried fenugreek leaves (ADFL), unprocessed fenugreek seeds (UFS), germinated fenugreek seeds (GFS), soaked fenugreek seeds (SFS), and boiled fenugreek seeds (BFS) against three cancerous cells from hepatic (HepG2), colon (HCT-116), and breast (MCF-7) cancer cell lines in comparison to normal kidney cell lines (VERO) using MTT assays. AGE, comet assay, RT-PCR, and ELISA methods were used to study the ability of each studied sample on DNA damage and regulation of pro- and anti-apoptotic genes, by exposing p53- caspase-3, Bax, and Bcl-2 of HepG2, HCT-116, MCF-7, and VERO cells to IC_50_ doses of ethanolic crude extracts of each tested sample. Finally, a Western blot assay was used to measure the protein-expressing levels of both IKK-α and IKK-β protein kinases in each cancerous and normal cell line exposed to the IC_50_ doses of the studied samples.

## 2. Materials and Methods

### 2.1. Materials

#### 2.1.1. Chemicals 

All RPMI 1640 culture media, glutamine, FBS, penicillin, streptomycin, trypsin, primers for p53, caspase-3, Bcl-2, Bax, GAPDH, solvents, dyes, and chemicals were purchased from Sigma-Aldrich, St. Louis, MO, USA. Primary antibodies included TNF-α, 8-OHDG, Cas-3, GAPDH, IKK-α, and IKK-β, and horseradish peroxidase-conjugated secondary antibodies were obtained from Santa Cruz Biotechnology, (Dallas, TX, USA). Western blot kits were purchased from Amersham, Buckinghamshire, UK.

#### 2.1.2. Cell Lines and Culturing Conditions

All cell lines were obtained from the American Type Culture Collection (ATCC, Manassas, VA, USA). Anti-proliferative activity was investigated in vitro against three human cancerous cell lines, including the human hepatic (HepG2), breast (MCF-7), and colon (HCT-116) carcinoma cell lines, and normal human kidney cell line (VERO), and determined by the Bioassay—Cell Culture Laboratory, National Research Centre. All cell lines were cultured in RPMI 1640 medium supplemented with 2 mM L-glutamine, 10% FBS, and 1% penicillin/streptomycin. Then, sub-confluent cultures (70–80%) were trypsinized (trypsin 0.05%/0.53 mM EDTA) and split depending on the seeding ratio.

#### 2.1.3. Plant Materials and Preparation

##### Air-Dried Fenugreek Leaves (ADFL)

Fresh and healthy leaves of fenugreek (*Trigonella foenum-graecum* Linn.) were purchased from the local market in Egypt in the month of December 2019 and identified at the Faculty of Science, Cairo University. The leaves were washed thoroughly with tap water, and the surface water was removed by air-drying under shade for 15 days. Leaves were subsequently dried in a hot air oven at 50 °C for 4 h, homogenized to a fine powder, and then stored at 4 °C.

##### Fenugreek Seeds

Two kilograms of fenugreek seeds were purchased from the local market in Egypt, identified at the Faculty of Science, Cairo University, and divided into four groups as follows:

(a) Untreated Fenugreek Seeds (UFS)

Five hundred grams of seeds were cleaned manually to remove dust and foreign particles, crushed into a fine powder flour with the help of Moulinex blender LM 241, and sieved through a 0.5 mm mesh size. The powdered flour was kept at 4 °C to prevent changes until further analysis [11].

(b) Soaked Fenugreek Seeds (SFS)

Five hundred grams of untreated seeds were soaked in tap water at the ratio of 1:5 (*w/v*) for 12 h at room temperature. After removing the soaking water, the seeds were air-dried in shade for 5 days, followed by hot air oven-drying at 50 °C for 4 h in a conventional oven, and stored at 4 °C [12].

(c) Germinated Fenugreek Seeds (GFS)

Five hundred grams of untreated seeds were soaked in tap water at the ratio of 1:5 (*w/v*) for 12 h at room temperature. After removing the soaking water, seeds were kept in the dark for germination (tied in a cotton cloth) at 20 °C for 60 h in darkness. After harvesting the sprouts, they were air-dried for 5 days, followed by hot air oven-drying at 50 °C for 4 h in a conventional oven, and stored at 4 °C [12].

(d) Boiled Fenugreek Seeds (BFS)

Five hundred grams of untreated seeds were put in 2 L beaker containing 1250 mL tap H_2_O (1:5 *w/v*). The sample was boiled on a hot plate for 10 min, and then, after the water was discarded, the boiled seeds were air-dried in shade for 5 days, followed by hot air oven-drying at 50 °C for 4 h in a conventional oven, and stored at 4 °C [13].

### 2.2. Methods

#### 2.2.1. Extraction Procedure of Samples

One hundred grams of each sample were extracted with 1 L (1:10 *w/v*) 80% ethanol in distilled H_2_O through sonication for 60 min. Extraction was repeated three times. After filtration, an extract of each sample was condensed until dry (resulting in crude extracts) by use of a rotary evaporator at 40 °C. A known weight of each crude extract was dissolved in appropriate volume of dimethyl sulfoxide (DMSO) and stored at −80 °C until further analysis [12].

#### 2.2.2. In Vitro Anti-Proliferative Activity (MTT Assay)

The anti-proliferative property of crude extracts of the selected samples against each cancerous and normal cell line was examined using 3-(4,5-dimethylthiazol-2-yl)-2,5-diphenyltetrazolium bromide (MTT) assay, according to Mosmann [14].

#### 2.2.3. Analysis of DNA Fragmentation Using Agarose Gel Electrophoresis (AGE) 

The qualitative method for assessing cell death by detecting DNA fragments using AGE was performed according to Kasibhatla et al. [15].

#### 2.2.4. Evaluation of DNA Damage Using Comet Assay

The method of Kent et al. [16] was followed to evaluate the comet moment as a measure of DNA damage in the HepG2 and MCF-7 cancerous cell lines, which were exposed to IC_50_ of each studied sample. 

#### 2.2.5. Real Time-PCR (RT-PCR)

Gene expression of pro-apoptotic genes (including p53, Cas-3, Bcl-2) and Bax, an anti-apoptotic gene, were tested against HepG2, HCT-116, MCF-7, and VERO cells exposed to IC_50_ doses of the tested samples for 24 h, according to Robert [17]. Raw data were analyzed using the Rotor-Gene^®^cycler software 2.1 (Qiagan Gmbh, Düsseldorf, Germany) to calculate the threshold cycle (Ct) using the second derivative maximum. The fold change value for each gene was determined after normalization to the expression levels of GAPDH as an HK gene, which was calculated using the equation 2^−∆∆Ct^.

#### 2.2.6. Apoptosis Enzyme-Linked Immunosorbent Assay (ELISA)

Quantitative analysis of TNF-α, 8-OHDG, and Cas-3 antibodies using ELISA technique was investigated according to the method of Khalil [18]. Each cell of HepG2 and MCF-7 exposed to IC_50_ of crude extracts of each studied sample was seeded at a density of 2 × 10^4^/well in a 96-well plate and incubated for 24 h. Absorbance was measured using an ELISA reader (Jenway Spectrophotometer, London, UK). Determination was performed in triplicate, and standard deviation was determined.

#### 2.2.7. Western Blotting Analysis

The Western blot for IKK-α and IKK-β primary antibodies was performed according to the method of Morgia [19]. The expression of GAPDH was used as a normalization control for protein loading. The corresponding relative density of IKK-α and IKK-β bands was calculated via Quantity Onesoftware.

#### 2.2.8. Statistical Analysis

All assays used in this work were evaluated in triplicate, and the data obtained were represented by the mean ± standard deviation (SD). Statistical Analysis Software (SAS 9.1) was applied for the statistical analysis of data, and IC_50_ was calculated by using GraphPad prism 7. One-way analysis of variance (ANOVA) was used to analyze the difference between groups by applying the least significant difference (LSD) test with a 5% level of significance (*p* < 0.05).

## 3. Results

### 3.1. Anti-Proliferative Activity of Household-Processed Fenugreek Leaves and Seeds

The impacts of household-processed fenugreek leaves and seeds on the cell proliferation of HepG2, HCT-116, MCF-7, and VERO cell lines exposed to serial concentrations of the tested samples are presented in Table 1, Table 2, Table 3 and Table 4. As shown in Table 1, the air-dried fenugreek leaves sample (ADFL) was the most potent selected sample according to its effectiveness against the HepG2 cell line (90%) at 500 µg mL^−1^. In contrast, the household processing of fenugreek seeds at 1000 µg mL^−1^ caused a significant decrease in the cell proliferation of the HepG2 cells, and BFS had the lowest cytotoxicity (56%). The IC_50_ values were 220 µg mL^−1^ for ADFL and 320 µg mL^−1^ for UFS, although these values increased after household treatments. 

Furthermore, the impact of the tested samples on the human colon cancer cell line (HCT-116) is represented in Table 2, and the data show the same trend as HepG2. The ADFL sample at 500 µg mL^−1^ also had the highest anti-proliferative activity (95%) against the HCT-116 cell line, and the germinated (GFS), soaked (SFS), and boiled (BFS) fenugreek seeds reduced the activity compared to the untreated seeds (UFS). The IC_50_ values were 240 µg mL^−1^ for ADFL and 275 µg mL^−1^ for UFS, although these values increased after household treatments. 

On the contrary, as shown in Table 3, at 1000 µg mL^−1^, both the untreated and germinated fenugreek seeds (UFS and GFS) had the highest potential cytotoxicity against the MCF-7 (95%), compared to the other tested samples. ADFL had 92% cytotoxicity at 1000 µg mL^−1^, SFS had 90% at 2000 µg mL^−1^, and BFS had 95% at 2000 µg mL^−1^. The IC_50_ values of both UFS and GFS were 500 and 620 µg mL^−1^, respectively. In addition, the soaking and boiling treatments of seeds at 1000 µg mL^−1^ caused a significant decline (91% and 56%, respectively) in the cell proliferation of MCF-7. Protodioscin, a steroidal saponin of fenugreek, had an inhibitory impact on the leukemic cell line (HL-60) and a weak inhibitory effect on the growth of the gastric cell line (KATO-III) [20]. 

Finally, the effect of the tested samples on the cell proliferation of the normal kidney cell line (VERO) can be observed in Table 4. According to the results, the IC_50_ (µg mL^−1^) values of ADFL, UFS, and GFS were 1660, 1840, and 1940 µg mL^−1^, respectively. However, the soaking and boiling treatments had no effects on the normal VERO cell line.

### 3.2. DNA Fragmentation Using Agarose Gel Electrophoresis (AGE) 

**Figure 1:**
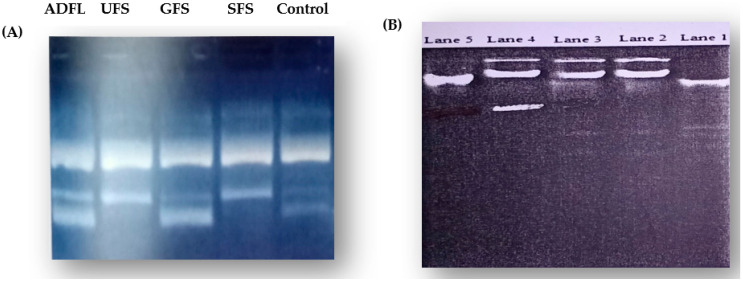
(**A**) DNA fragmentation analysis using agarose gel electrophoresis (AGE). Control: untreated cell line of HepG2, ADFL: HepG2 treated with IC_50_ of air-dried fenugreek seeds, UFS: HepG2 treated with IC_50_ of unprocessed fenugreek seeds, GFS: HepG2 treated with IC_50_ of germinated fenugreek seeds, SFS: HepG2 treated with IC_50_ of soaked fenugreek seeds. (**B**) DNA fragmentation using agarose gel electrophoresis. Lane 5: untreated cell line (control) of MCF-7, Lane 4: MCF-7 treated with IC_50_ dose of air-dried fenugreek seed (ADFL) sample, Lane 3: MCF-7 treated with IC_50_ dose of unprocessed fenugreek seed (UFS) sample, Lane 2: MCF-7 treated with IC_50_ dose of germinated fenugreek seed (GFS) sample, Lane 4: MCF-7 treated with IC_50_ dose of soaked fenugreek seed (SFS) sample.

Agarose gel electrophoresis (AGE) was used to evaluate the DNA fragmentation activity of the tested samples to indicate whether or not the action of the extract is associated with apoptosis. DNA fragmentation of the HepG2 and MCF-7 cancer cell lines treated with the IC_50_ doses of each tested sample can be observed in Figure 1.

As shown in Figure 1A,B, the formation of a DNA ladder in the HepG2 and MCF-7 cell lines treated with IC_50_ doses of each studied sample was observed. The treatment of the HepG2 and MCF-7 cell lines with the IC_50_ doses of the household-processed fenugreek leaves and seeds (ADFL, UFS, GFS, and SFS, respectively) caused the fragmentation of DNA in each cancer cell line compared to the untreated control cell lines and the cells exposed to the IC_50_ of the BFS sample. Therefore, the air-dried fenugreek leaves (ADFL) and unprocessed and germinated fenugreek seeds (UFS and GFS, respectively) induced DNA fragmentation and, thus, apoptosis in both HepG2 and MCF-7 cancer cell lines.

### 3.3. DNA Damage Using Comet Assay

The DNA damage of the HepG2 and MCF-7 cancer cell lines was observed by using single-cell gel electrophoresis (SCGE) (Comet assay), which is able to detect DNA strand breaks (SBs) and oxidative DNA damage in cultured cell lines.

As presented in Table 5, the comet percentages of the untreated cell line (control) were 9.21 and 8.81% for the HepG2 and MCF-7 cell line, respectively. A higher percentage resulted in the cell lines treated with the IC_50_ doses of ADFL, UFS, GFS, SFS, and BFS, which were 13.01, 12.91, 11.41, 10.00, and 9.31%, respectively, for HepG2 cell lines, and were 12.27, 11.10, 9.89, 9.66, and 8.77%, respectively, for MCF-7 cell lines. 

The values of head diameters are also observable in Table 5, and the data show that there are no significant differences between the HepG2 and MCF-7 cell lines treated with IC_50_ doses of the studied samples. The head diameters of the untreated HepG2 and MCF-7 cell lines were 17.61 and 17.15 px, respectively. 

The percentages of DNA values in the heads of the untreated HepG2 and MCF-7 cell lines (control) were 84.00 and 81.51%, respectively. In contrast, the HepG2 cell lines exposed to the IC_50_ doses of the tested samples had a decrease in DNA % in the head (80.01% for ADFL, 80.51% for UFS, 81.11% for GFS, and 83.69% for SFS), except in the BFS sample (84.01%), which was not significantly different compared to the control sample. The values were 76.01, 78.00, 79.00, 81.04, and 81.48% for the MCF-7 cell lines treated with ADFL, UFS, GFS, SFS, and BFS, respectively.

The data in Table 5 also demonstrate that the value of the tail length of the untreated HepG2 cell line was 3.82 px and increased after exposure to the IC_50_ of ADFL (4.00 px), UFS (3.94 px), and GFS (3.91 px), but was not significantly changed after treatment with either SFS (3.85) or BFS (3.81) samples. On the other hand, the tail length value of the untreated control cell line of MCF-7 was 3.80 px, and increased to 4.01, 3.98, 3.90, and 3.85 px after exposure to ADFL, UFS, GFS, and SFS, respectively, with the exception of the BFS sample (3.81 px).

Furthermore, the percentages of DNA in the head were found to be 22.17% for ADFL, 21.00% for UFS, 19.41% for GFS, 18.12% for SFS, and, lastly, 18.00% for BFS relative to the untreated control HepG2 cell lines. In addition, the untreated MCF-7 cell line (control) had a DNA% in the head of 18.20%, which was also raised to 23.21, 22.11, 20.00, and 18.41% for ADFL, UFS, GFS, and SFS, respectively, but did not vary with the BFS sample (18.21%). 

Lastly, the tail moment values of DNA of the untreated (control) HepG2 and MCF-7 cell lines were 1.02 and 0.93, respectively. These values were higher after the exposure of HepG2 cell lines to ADFL (1.16), UFS (1.14), GFS (1.10), and SFS (1.07), but did not differ from the BFS sample (1.01). The untreated control MCF-7 cell lines had a tail moment of 0.93 and increased after treatment with the IC_50_ of ADFL (1.10), UFS (1.07), GFS (1.01), and SFS (0.96), but not with the BFS sample (0.94). 

### 3.4. Gene Expression of Apoptotic Marker Genes Using RT-PCR

**Figure 2:**
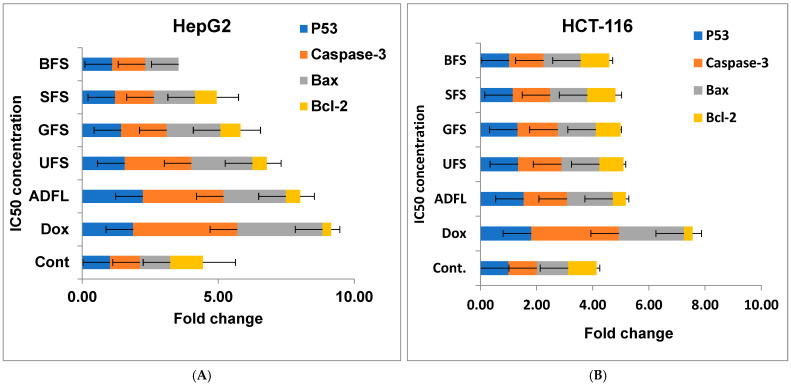

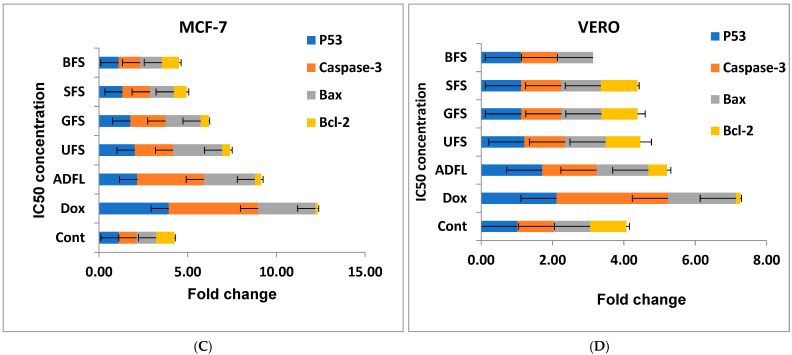
(**A**) Fold change in the level of mRNA expression of various apoptosis genes via RT-PCR in human hepatic carcinoma cell lines (HepG2) exposed to IC_50_ (µg mL^−1^) doses of doxorubicin (Dox) as standard drug, air-dried fenugreek leaves (ADFL), untreated fenugreek seeds (UFS), germinated fenugreek seeds (GFS), soaked fenugreek seeds (SFS), and boiled fenugreek seeds (BFS) for 24 h. (**B**) Fold change in the level of mRNA expression of various apoptosis genes via RT-PCR in human colon carcinoma cell lines (HCT-116) exposed to IC_50_ (µg mL^−1^) doses of doxorubicin (Dox) as standard drug, ADFL, UFS, GFS, SFS, and BFS for 24 h. (**C**) Fold change in the level of mRNA expression of various apoptosis genes via RT-PCR in human breast carcinoma cell lines (MCF-7) exposed to IC_50_ (µg mL^−1^) doses of doxorubicin (Dox) as standard drug, ADFL, UFS, GFS, SFS, and BFS for 24 h. (**D**) Fold change in the level of mRNA expression of various apoptosis genes via RT-PCR in normal kidney cell lines (VERO) exposed to IC_50_ (µg mL^−1^) doses of doxorubicin (Dox) as standard drug, ADFL, UFS, GFS, SFS, and BFS for 24 h. The data provided are mean ± standard deviation from three separate experiments. *p* < 0.05 versus control.

The gene expression of the pro-apoptotic genes involved either p53, Caspase-3 (Cas-3), or Bax, as well as the anti-apoptotic Bcl-2 gene, against HepG2, HCT-116, MCF-7, and VERO cell lines treated with IC_50_ doses of the selected sample, is summarized in Figure 2. The results are expressed as the ratio of the reference gene (housekeeping gene/HKG/GAPDH) to the target genes (including p53, Cas-3, Bax, and Bcl-2) by fold change.

According to Figure 2A, hepatic cancer cell lines (HepG2) treated with the IC_50_ doses of ADFL, UFS, GFS, SFS, and BFS are compared to the untreated cell lines (control). The results showed that air-dried fenugreek leaves (ADFL) were the most potent sample, which significantly up-regulated p53, Cas-3, and Bax (at 2.23, 2.97, and 2.29 fold change, respectively) and down-regulated Bcl-2 (at a 0.52 fold change) compared to untreated cell lines. However, the standard drug (doxorubicin) caused an up-regulation of the studied pro-apoptotic genes, as follows: 1.87 for p53, 3.82 for Cas-3, and 3.11 fold change for Bax genes, and a down-regulation of the Bcl-2 gene (at a 0.32 fold change). In addition, each of the UFS, GFS, SFS, and BFS samples significantly increased the up-regulation of p53 (at 1.56, 1.43, 1.21, and 1.10 fold change, respectively), Cas-3 (at 2.45, 1.67, 1.42, and 1.22 fold change, respectively), and Bax (at 2.24, 1.98, 1.51, and 1.22 fold change, respectively) genes relative to the untreated control cell lines. 

On the other hand, human colon cancer cell lines (HCT-116) exposed to IC_50_ doses of ADFL, UFS, GFS, SFS, and BFS compared to both the control cell line and the other samples that were treated with doxorubicin were also used to study the apoptosis-inducing property of the tested samples (Figure 2B). Data showed that ADFL was the most effective sample in up-regulating p53, Cas-3, and Bax (at 1.54, 1.54, and 1.63 fold change, respectively) and down-regulating the Bcl-2 gene (at a 0.46 fold change) compared to untreated cells. The standard drug (doxorubicin) demonstrated an up-regulation of the studied pro-apoptotic genes as follows: 1.81 for p53, 3.12 for Cas-3, and a 2.31 fold change for Bax genes, and a down-regulation of the Bcl-2 gene (at a 0.31 fold change). Whereas, the UFS, GFS, SFS, and BFS samples induced the up-regulation of p53 (at 1.34, 1.32, 1.15, and 1.02 fold change, respectively), Cas-3 (at 1.54, 1.43, 1.33, and 1.23 fold change, respectively), and Bax (at 1.36, 1.36, 1.32, and 1.32 fold change, respectively) genes and the down-regulation of the Bcl-2 gene (at 0.85, 0.88, 1.00, and 1.01 fold change) relative to the standard drug doxorubicin.

Furthermore, breast cancer cell lines (MCF-7) treated with IC_50_ in each studied sample were compared to the untreated cell lines, and the other samples treated with doxorubicin were also used to evaluate the apoptotic-inducing activity of the tested samples (Figure 2C). Findings showed that air-dried fenugreek leaves (ADFL) exhibited the highest up-regulation of p53, Cas-3, and Bax (at 2.15, 3.76, and 2.88 fold change, respectively) and down-regulation of the Bcl-2 gene (at a 0.35 fold change) in comparison to untreated cells. The exposure of cell lines to doxorubicin as a standard drug induced the up-regulation of the studied pro-apoptotic genes as follows: 3.93 for p53, 5.03 for Cas-3, and 3.23 fold changes for Bax genes, and down-regulated the Bcl-2 gene (at a 0.12 fold change). However, the UFS, GFS, SFS, and BFS samples enhanced the up-regulation of p53 (at 2.01, 1.76, 1.32, and 1.10 fold change, respectively), Cas-3 (at 2.17, 1.98, 1.54, and 1.22 fold change, respectively), and Bax (at 2.77, 1.99, 1.35, and 1.22 fold change, respectively) genes and the down-regulation of the Bcl-2 gene (at 0.44, 0.46, 0.72, and 0.97 fold change, respectively), as compared to doxorubicin.

Finally, a normal kidney cell line (VERO) exposed to the IC_50_ concentration of each studied sample was also used to study the apoptosis-inducing properties of the tested samples (Figure 2D). The data showed that air-dried fenugreek leaves (ADFL) were the most effective sample, which up-regulated p53, Cas-3, and Bax (at 1.71, 1.52, and 1.45 fold change, respectively) and down-regulated the Bcl-2 gene (at a 0.52 fold change) compared to untreated cells. Doxorubicin up-regulated the studied genes as follows: 2.11 for p53, 3.12 for Cas-3, and a 1.90 fold change for Bax genes, and down-regulated the Bcl-2 gene (at a 0.12 fold change). Whereas, each UFS, GFS, SFS, and BFS sample induced the up-regulation of p53 (at 1.21, 1.12, 1.12, and 1.11 fold change, respectively), Cas-3 (at 1.14, 1.13, 1.12, and 1.01 fold change, respectively), and Bax (at 1.13, 1.12, 1.11, and 1.01 fold change, respectively) genes and the down-regulation of the Bcl-2 gene (at 0.97, 1.01, 1.02, and 1.12 fold change) relative to untreated cell lines.

According to these findings, we summarized that the treatment of hepatic, colon, and breast cancer cell lines exposed to IC_50_ doses of household-processed fenugreek leaves and seeds involved ADFL, UFS, GFS, SFS, and BFS, which enhanced the up-regulation of the studied pro-apoptotic p53, Cas-3, and Bax genes and down-regulation of anti-apoptotic Bcl-2 genes, which induced apoptosis of the studied cell lines, then decreased the tumor growth and cell proliferation.

### 3.5. Quantitative Gene Expression of Tumor Markers Using ELISA Assay

**Figure 3:**
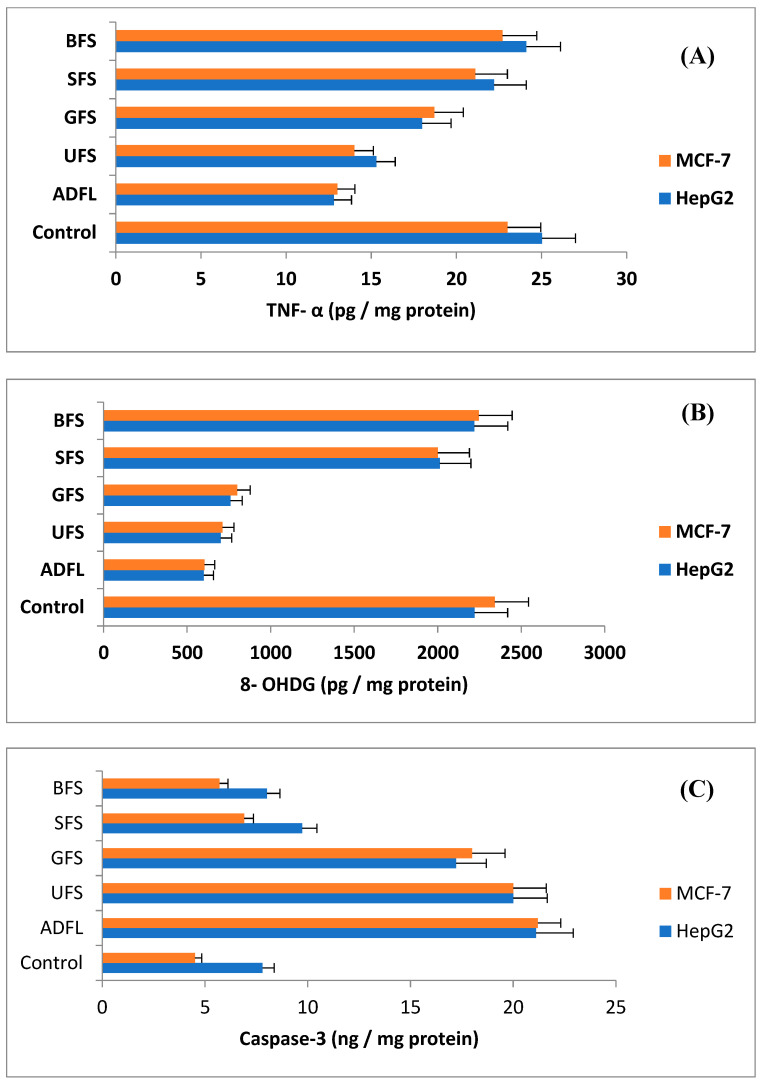
(**A**) (ELISA): The relative expression of tumor necrosis factor alpha (TNF-α) of each untreated (Control) HepG2 and MCF-7 cancer cell lines and others treated with the IC_50_ doses of air-dried fenugreek leaves (ADFL), untreated fenugreek seeds (UFS), germinated fenugreek seeds (GFS), soaked fenugreek seeds (SFS), and boiled fenugreek seeds (BFS). (**B**) The relative expression of 8-hydroxy-2- deoxyguanosine in untreated HepG2 and MCF-7 cancer cell lines and those treated with the IC_50_ doses of ADFL, UFS, GFS, SFS, and BFS. (**C**) The relative expression of Cas-3 in untreated HepG2 and MCF-7 cancer cell lines and those treated with the IC_50_ doses of ADFL, UFS, GFS, SFS, and BFS.

Tumor necrosis factor alpha (TNF-α) is an inflammatory cytokine which is involved in immune function and is also proposed to play a role in metabolic disorders. The results presented in Figure 3A show that the decrease in TNF-α was significant with the household-processed samples compared to the unprocessed fenugreek seeds (UFS), except for boiled seeds (BFS). The results indicated that TNF-α values appeared to be 12.81 and 13.01, 15.03 and 14.01, 17.99 and 18.71, 22.21 and 21.11, and lastly, 24.11 and 22.71 pg mg^−1^ protein, respectively, for each HepG2 and MCF-7 cell lines treated with IC_50_ doses of ADFL, UFS, GFS, SFS, and BFS. This caused a significant decline up to 51 and 57%, 61 and 61%, 72 and 81%, 89 and 92%, and finally, 96 and 99%, respectively for the same tested samples. The control (untreated cell line) showed 25.03 and 23.00 pg mg^−1^ protein for each untreated HepG2 and MCF-7 cell line.

An 8-Hydroxy-2′-deoxyguanosine (8-OHDG) biomarker was evaluated to measure DNA damage. The results presented in Figure 3B are significant for all tested samples, especially the air-dried fenugreek leaves (ADFL), which had the most potent activity for reducing the 8-OHDG factor compared to the untreated cell line (control). The level of 8- OHDG factor in the hepatic (HepG2) and breast (MCF-7) cell lines appeared to be 600 and 604 pg/mg protein (for air-dried fenugreek leaves/ADFL), 700 and 712 pg/mg protein (for untreated fenugreek seeds/UFS), 760 and 800 pg/mg protein (germinated fenugreek seeds/GFS), 2012 and 2001 pg/mg protein (for soaked fenugreek seeds/SFS), and 2219 and 2246 pg/mg protein (for boiled fenugreek seeds/BFS), respectively, compared to the untreated, HepG2 and MCF-7, cell lines (2220 and 2342 pg/mg protein), respectively. These values showed percentages of 27 and 26% (ADFL), 32 and 30% (UFS), 34 and 34% (GFS), 91 and 85% (SFS), and 100 and 96% (BFS) for HepG2 and MCF-7, respectively, in comparison with the untreated cell line (control).

The caspase-3 (Cas-3) factor is usually used to evaluate the apoptotic cascade. This biomarker factor was activated and increased after exposure to the HepG2 and MCF-7 cell lines to the studied samples, with the exception of the boiled fenugreek seeds (BFS). According to the presented data in Figure 3C, the impacts of the examined samples (ADFL, UFS, GFS, SFS, and BFS) on the levels of Cas-3 factor were observed to be 21.11 and 21.20, 20.00 and 20.00, 17.22 and 18.00, 9.73 and 6.91, and lastly, 8.01 and 5.71 ng/mg protein, respectively, which were calculated as the percentages 270 and 470%, 256 and 443%, 221 and 399%, 125 and 153%, and finally, 103 and 127% relative to the untreated control sample, which showed the values of 7.80 and 4.51 ng/mg protein for the HepG2 and MCF-7 cell lines, respectively.

### 3.6. Protein Expression of IKK-α and IKK-β 

**Figure 4:**
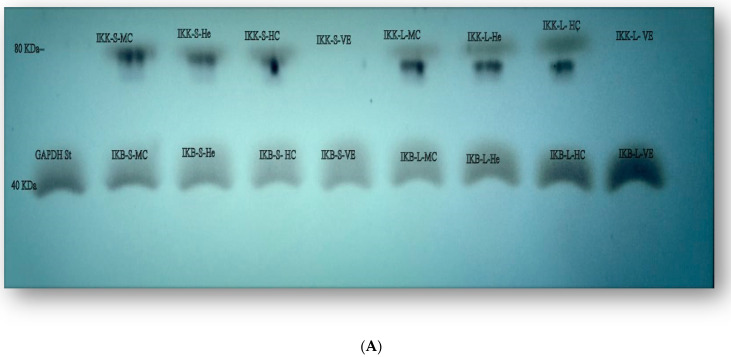

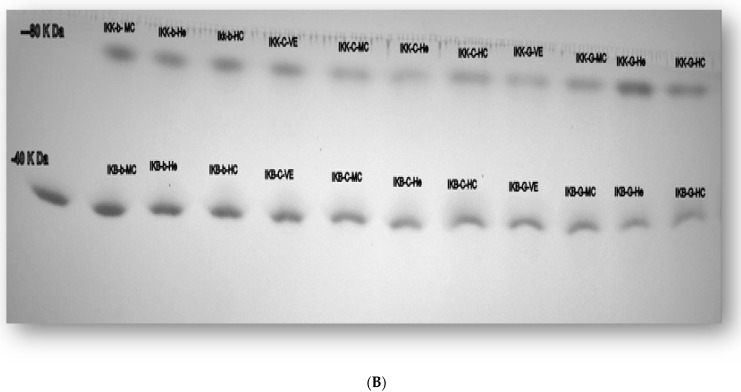
(**A**) IKK-α and IKK-β images for boiled (b), unprocessed (C), and germinated fenugreek seeds (G) on VERO (VE), MCF-7 (MC), HepG2 (He), and HCT-116 (HC) cell lines. The image shows that IKK separated at 80 K Da, and IKB separated around 40 K Da. (**B**) IKK and IKB images for GAPDH, soaked fenugreek seeds (S) and air-dried fenugreek leaves (L) on VERO (VE), MCF-7 (MC), HepG2 (He), and HCT-116 (HC) cell lines, the image also shows that IKK separated at 80 K Da, and IKB separated around 40 K Da.

To further confirm the results of the IKK-α and IKK-β expressions, the signaling pathway was further investigated through a Western blotting assay (Figure 4A,B and Table 6 and Table 7). 

As observable in Table 6, the UFS, BFS, and GFS samples had the most activity on the expression levels of IKK-α in the HepG2, MCF-7 and HCT-116 cell lines, which were exposed to IC_50_ doses (66.45, 63.21, and 61.54% in HepG2, 66.88, 58.95, and 60.01% in MCF-7, and 61.85, 61.48, and 54.43% in HCT-116, respectively). Meanwhile, IKK-α expression levels in VERO cells were not detected in all studied samples, except for UFS (60.78%) and GFS (64.83%).

In contrast, the results shown in Table 7 demonstrate that both the ADFL and SFS samples had the most activity on the expression levels of IKK-β in the HepG2, MCF-7, and VERO cell lines which were exposed to IC_50_ doses (62.49 and 50.03% in HepG2, 46.57 and 44.56% in MCF-7, 44.56 and 100% in VERO, respectively). Meanwhile, IKK-β expression levels were been high in HCT-116 cells treated with SFS (46.90%), GFS (45.57%), and ADFL (44.56%), respectively.

## 4. Discussion

The fenugreek plant has had an important role for a long time in the treatment and prevention of diseases. Various studies have also proved many of these conventional applications and have observed the beneficial value of this plant and its capabilities in traditional medicine. However, the scientific information about the evaluation of the mechanism of action of this plant is not enough [21].

Finding naturally occurring chemo-preventive compounds has received more attention in recent years, especially those found in dietary and medicinal plants due to their bioactive components [22,23]. 

These findings are in agreement with Raju [24] and Thirunavukkarasu [25], who found that diosgenin, a steroidal saponin in the fenugreek plant, displayed anti-proliferative properties by blocking the proliferation of human colon cancer cells (HT-29) and induced apoptosis by exhibiting the activation of caspase-3 and inhibition of Bcl-2. 

The majority of these bioactive chemicals inhibit cell cycle progression and cause apoptotic cell death to carry out their cancer-chemotherapeutic activities. As a result, the induction of apoptosis in tumor cells has evolved into a marker of the tumor treatment response when using a bioactive chemical derived from plants to lower and manage cancer-related mortality in humans [26,27]. Therefore, there is a huge need to look for innovative alternative agents for the prevention and treatment of hepatic, breast, and colon cancers.

In this work, household processes were been involved in the air-drying of fenugreek leaves for preserving the leaves from spoilage resulting from the moisture content of fresh leaves, and soaking, germination, and boiling of raw fenugreek seeds to reduce the bitter taste, due to the presence of saponins, and specific smell, due to the presence of alkaloids and volatile oils, which limit their usability and acceptability in food industries. This study aimed to evaluate the anti-proliferative activity of air-dried fenugreek leaves (ADFL), unprocessed fenugreek seeds (UFS), germinated fenugreek seeds (GFS), soaked fenugreek seeds (SFS), and boiled fenugreek seeds (BFS) against three cancerous cells involved hepatic (HepG2), colon (HCT-116), and breast (MCF-7) cells in comparison to normal kidney cell lines by using an MTT assay.

A typical genetic change linked to cancer is the mutation of the tumor suppressor p53, which inactivates 50% of human malignancies [28]. A complex network of posttranslational changes regulates the activity, stability, and molecular interactions of the p53 protein, which controls apoptosis and cell cycle arrest [29]. Late in the process of hepatic carcinogenesis, HCC develops p53 mutation [30]. However, there is not much information available on the connection between p53 WT expression and differentiation grade. An alternate strategy for the treatment of HCC is the intervention to reinstate wild-type p53 activity. The role of p53 in the pathogenesis, development, therapeutic effects, diagnosis, treatment, and prognosis of HCC has recently been the subject of numerous investigations [31,32,33].

DNA damage is one of the most well-known effects of oxidative stress. Biomarkers for nucleic acid oxidation are frequently utilized to evaluate this damage [34]. The most well-known and often-used biomarker of nucleic acid oxidation is 8-hydroxy-20 deoxyguanosine (8-OHdG), whose formation by free radicals was originally noted in 1984 [20]. This biomarker has been used to assess oxidative stress brought on by exposure to a variety of agents in the workplace and in the environment [35]; these agents include fine particulates [36], polycyclic aromatic hydrocarbons [37], chromium [38], traffic exhausts [39], air pollution, and many others.

Through the inhibition of Akt activity, diosgenin reduced TNF-induced NF-κB activation, IκB-α kinase activation, IκB-α phosphorylation, IκB degradation, p65 (RELA) phosphorylation, and p65 nuclear translocation. Diosgenin also inhibited the TNF-induced production of genes related to cell proliferation (cyclin D1, COX-2, and c-myc), anti-apoptosis (IAP1, Bcl-2, Bcl-XL, Bfl-1/A1, and cFLIP), and invasion (matrix metalloproteinase (MMP)-9). Additionally, TNF and chemotherapeutic medicines such as paclitaxel and doxorubicin that promote apoptosis were potentiated by diosgenin [40].

## 5. Conclusions

The primary public health issue across the globe is cancer. The identification of naturally occurring chemo-preventive compounds has recently received increased interest, particularly those found in dietary and medicinal plants because of their bioactive components. The majority of these bioactive chemicals inhibit cell cycle progression and cause apoptotic cell death to carry out their cancer-chemotherapeutic activities. As a result, the induction of apoptosis in tumor cells has evolved into a marker of the tumor treatment response when using a bioactive substance produced from plants to lower and manage cancer-related mortality in humans. When compared to the VERO cell line, air-dried fenugreek leaves, raw fenugreek seeds, and germinated fenugreek seeds had the most anti-proliferative and apoptosis-inducing effects on each of the human HepG2, MCF-7, and HCT-116 cell lines. So, these crude extracts can be used in the future for developing new effective natural drugs for the treatment of hepatocellular, breast, and colon carcinomas.

## Figures and Tables

**Table 1:** Anti-proliferative activity and IC_50_ (µg mL^−1^) values of ethanolic crude extracts of air-dried fenugreek leaves (ADFL), untreated fenugreek seeds (UFS), germinated fenugreek seeds (GFS), soaked fenugreek seeds (SFS), and boiled fenugreek seeds (BFS) against human hepatic carcinoma cell line (HepG2).

Cytotoxicity % (Mean ± SD)						
Concentration (µg mL^−1^)						IC_50_ (µg mL^−1^)
	100	200	300	400	500	
**ADFL**	23 ± 1.7 ^a^	46 ± 2.9 ^a^	70 ± 4.6 ^a^	80 ± 5.2 ^a^	90 ± 5.6 ^a^	220
**UFS**	16 ± 1.0 ^b^	32 ± 2.0 ^b^	48 ± 3.1 ^b^	61 ± 3.8 ^b^	80 ± 5.1 ^b^	320
**GFS**	12 ± 1.0 ^c^	24 ± 1.0 ^c^	36 ± 2.0 ^c^	43 ± 2.6 ^c^	60 ± 4.0 ^c^	420
	**200**	**400**	**600**	**800**	**1000**	**IC_50_** **(µg mL^−1^)**
**SFS**	20 ± 1.6 ^a^	40 ± 2.7 ^a^	60 ± 4.1 ^a^	82 ± 5.1 ^a^	91 ± 5.7 ^a^	500
**BFS**	11 ± 0.6 ^b^	22 ± 1.3 ^b^	33 ± 2.0 ^b^	44 ± 3.0 ^b^	56 ± 3.7 ^b^	900

Each value is mean ± SD. Values in the same column followed by the same letter are not significant in difference at *p* ≤ 0.05.

**Table 2:** Anti-proliferative activity and IC_50_ (µg mL^−1^) values of ethanolic crude extracts of each air-dried fenugreek leaves (ADFL), untreated fenugreek seeds (UFS), germinated fenugreek seeds (GFS), soaked fenugreek seeds (SFS), and boiled fenugreek seeds (BFS) against colon carcinoma cell line (HCT-116).

Cytotoxicity % (Mean ± SD *)						
Concentration (µg mL^−1^)						IC_50_ (µg mL^−1^)
	100	200	300	400	500	
**ADFL**	20 ± 1.2 ^b^	40 ± 2.6 ^a^	61 ± 4.1 ^a^	82 ± 5.1 ^a^	95 ± 5.6 ^a^	240
**UFS**	25 ± 1.4 ^a^	41 ± 2.8 ^a^	55 ± 3.3 ^b^	68 ± 4.1 ^b^	86 ± 5.3 ^b^	275
**GFS**	12 ± 0.8 ^c^	25 ± 1.7 ^c^	35 ± 2.1 ^c^	44 ± 3.0 ^a^	61 ± 4.0 ^c^	410
	**200**	**400**	**600**	**800**	**1000**	**IC_50_** **(µg mL^−1^)**
**SFS**	20 ± 1.4 ^a^	40 ± 2.8 ^a^	60 ± 4.0 ^a^	79 ± 4.1 ^a^	98 ± 5.1 ^a^	500
**BFS**	12 ± 0.7 ^b^	24 ± 1.5 ^b^	35 ± 2.1 ^b^	47 ± 2.9 ^b^	59 ± 3.1 ^b^	850

* Each value is mean ± SD. Values in the same column followed by the same letter are not significant in difference at *p* ≤ 0.05.

**Table 3:** Anti-proliferative activity (%) and IC_50_ (µg mL^−1^) values of ethanolic crude extracts of air-dried fenugreek leaves (ADFL), untreated fenugreek seeds (UFS), germinated fenugreek seeds (GFS), soaked fenugreek seeds (SFS), and boiled fenugreek seeds (BFS) against breast carcinoma cell line (MCF-7).

Cytotoxicity % (Mean ± SD *)						
Concentration (µg mL^−1^)						IC_50_ (µg mL^−1^)
	200	400	600	800	1000	
**ADFL**	18 ± 1.01 ^a^	44 ± 3.00 ^a^	60 ± 4.00 ^a^	74 ± 4.90 ^b^	92 ± 5.80 ^b^	480
**UFS**	18 ± 1.00 ^a^	40 ± 2.80 ^b^	60 ± 3.90 ^a^	78 ± 4.92 ^a^	95 ± 5.70 ^a^	500
**GFS**	16 ± 2.01 ^a^	31 ± 1.99 ^c^	49 ± 2.17 ^b^	60 ± 3.33 ^c^	95 ± 5.41 ^a^	620
	**400**	**800**	**1200**	**1600**	**2000**	**IC_50_** **(µg mL^−1^)**
**SFS**	21 ± 1.50 ^a^	40 ± 3.00 ^a^	61 ± 4.10 ^a^	80 ± 5.10 ^a^	90 ± 5.80 ^b^	980
**BFS**	19 ± 1.70 ^b^	38 ± 2.20 ^b^	57 ± 3.06 ^b^	76 ± 5.00 ^b^	95 ± 5.10 ^a^	1060

* Each value is mean ± SD. Values in the same column followed by the same letter are not significant in difference at *p* ≤ 0.05.

**Table 4:** Anti-proliferative activity (%) and IC_50_ (µg mL^−1^) values of ethanolic crude extracts of air-dried fenugreek leaves (ADFL), untreated fenugreek seeds (UFS), germinated fenugreek seeds (GFS), soaked fenugreek seeds (SFS), and boiled fenugreek seeds (BFS) against normal human kidney cell line (VERO).

Cytotoxicity % (Mean ± SD *)						
Concentration (µg mL^−1^)						IC_50_ (µg mL^−1^)
	400	800	1200	1600	2000	
**ADFL**	0	17 ± 1.11 ^a^	32 ± 2.13 ^a^	47 ± 2.97 ^a^	59 ± 3.11 ^a^	1660 ^c^
**UFS**	0	12 ± 1.20 ^b^	28 ± 1.56 ^b^	43 ± 2.51 ^b^	55 ± 3.00 ^ab^	1840 ^b^
**GFS**	0	0	12 ± 1.00 ^c^	33 ± 1.73 ^c^	51 ± 4.00 ^b^	1940 ^a^
**SFS**	0	0	0	0	0	0
**BFS**	0	0	0	0	0	0

* Each value is mean ± SD. Values in the same column followed by the same letter are not significant in difference at *p* ≤ 0.05.

**Table 5:** DNA damage using Comet assay for the HepG2 and MCF-7 cell lines treated with the IC_5o_ (µg mL^−1^) doses of control: untreated cell line, and crude extracts of ADFL: air-dried fenugreek leaves, UFS: unprocessed fenugreek seeds, GFS: germinated fenugreek seeds, SFS: soaked fenugreek seeds, and BFS: boiled fenugreek seeds.

Mean ± SD *							
Cell Lines	Studied Samples	Comet %	Head Diameter (Px)	DNA % in Head	Tail Length (Px)	DNA % in Tail	Tail Moment
**HepG2**	**Control**	9.12 ± 0.86 ^c^	17.61 ± 1.21 ^a^	84.00 ± 6.66 ^a^	3.82 ± 0.21 ^b^	18.01 ± 1.67 ^c^	1.02 ± 0.10 ^b^
	**ADFL**	13.01 ± 1.01 ^a^	17.55 ± 1.11 ^a^	80.01 ± 7.11 ^b^	4.00 ± 0.24 ^a^	22.17 ± 1.96 ^a^	1.16 ± 0.11 ^a^
	**UFS**	12.91 ± 1.00 ^a^	17.51 ± 1.30 ^a^	80.51 ± 6.71 ^b^	3.94 ± 0.19 ^a^	21.00 ± 1.69 ^b^	1.14 ± 0.11 ^a^
	**GFS**	11.41 ± 0.98 ^b^	17.58 ± 1.23 ^a^	81.11 ± 5.98 ^b^	3.91 ± 0.30 ^a^	19.41 ± 1.43 ^bc^	1.10 ± 0.09 ^b^
	**SFS**	10.00 ± 0.86 ^c^	17.60 ± 1.31 ^a^	83.69 ± 7.49 ^ab^	3.85 ± 0.29 ^b^	18.12 ± 1.51 ^c^	1.07 ± 0.10 ^bc^
	**BFS**	9.31 ± 0.67 ^c^	17.62 ± 1.61 ^a^	84.01 ± 6.93 ^a^	3.81 ± 0.37 ^b^	18.00 ± 1.51 ^c^	1.01 ± 0.08 ^bc^
**MCF-7**	**Control**	8.81 ± 0.73 ^c^	17.15 ± 1.16 ^a^	81.51 ± 6.31 ^b^	3.80 ± 0.26 ^b^	18.20 ± 1.67 ^c^	0.93 ± 0.08 ^c^
	**ADFL**	12.27 ± 1.11 ^ab^	17.03 ± 1.20 ^a^	76.01 ± 5.14 ^c^	4.01 ± 0.30 ^a^	23.21 ± 2.01 ^a^	1.10 ± 0.09 ^b^
	**UFS**	11.10 ± 0.89 ^b^	17.06 ± 1.01 ^a^	78.00 ± 6.14 ^b^	3.98 ± 0.29 ^a^	22.11 ± 2.00 ^a^	1.07 ± 0.11 ^bc^
	**GFS**	9.89 ± 0.77 ^c^	17.10 ± 1.13 ^a^	79.00 ± 5.99 ^b^	3.90 ± 0.31 ^a^	20.00 ± 1.89 ^ab^	1.01 ± 0.09 ^bc^
	**SFS**	9.66 ± 0.78 ^c^	17.14 ± 1.22 ^a^	81.04 ± 7.11 ^b^	3.85 ± 0.32 ^ab^	18.41 ± 1.77 ^c^	0.96 ± 0.08 ^c^
	**BFS**	8.77 ± 0.69 ^c^	17.16 ± 1.42 ^a^	81.48 ± 7.12 ^b^	3.81 ± 0.30 ^b^	18.21 ± 1.61 ^c^	0.94 ± 0.09 ^c^

* Each value is mean ± SD. Values in the same column followed by the same letter are not significant in difference at (*p* ≤ 0.05) % at control (untreated cell line).

**Table 6:** Protein expression levels of IKK-α in HepG2, MCF-7, HCT-116, and VERO cell lines exposed to IC_50_ of air-dried fenugreek leaves (ADFL), unprocessed fenugreek seeds (UFS), germinated fenugreek seeds (GFS), soaked fenugreek seeds (SFS), and boiled fenugreek seeds (BFS).

	IKK-α							
	HepG2		MCF-7		HCT-116		VERO	
	Area	Band %	Area	Band %	Area	Band %	Area	Band %
**ADFL**	7020	37.51	12,012	53.43	6708	32.95	ND *	ND
**UFS**	7728	66.45	8188	66.88	6624	61.85	6348	60.78
**GFS**	4140	61.54	5428	60.01	4140	54.43	6164	64.83
**SFS**	11,232	49.97	12,012	55.44	10,608	53.10	ND	ND
**BFS**	6716	63.21	6624	58.95	5888	61.48	ND	ND

* ND = Not Detected.

**Table 7:** Protein expression levels of IKK-β in HepG2, MCF-7, HCT-116, and VERO cell lines exposed to IC_50_ of air-dried fenugreek leaves (ADFL), unprocessed fenugreek seeds (UFS), germinated fenugreek seeds (GFS), soaked fenugreek seeds (SFS), and boiled fenugreek seeds (BFS).

	IKK-β							
	HepG2		MCF-7		HCT-116		VERO	
	Area	Band %	Area	Band %	Area	Band %	Area	Band %
**ADFL**	12,168	62.49	12.480	46.57	12,792	67.05	12,792	100
**UFS**	5060	33.55	4692	33.13	5060	38.15	5336	39.22
**GFS**	3404	38.46	4232	39.99	3864	45.57	3772	35.17
**SFS**	13,884	50.03	11,544	44.56	12,636	46.90	14,040	100
**BFS**	4416	36.79	4876	41.05	4508	38.52	ND *	ND

* ND = Not Detected.

## Data Availability

Data generated or analyzed during the current study are available from the corresponding author and included in this published article.

## Abbreviations

**8-OHDG**	8-Hydroxy-2′-deoxyguanosine	**IAP1**	*Inhibitor of Apoptosis Protein 1*
**ADFL**	Air-dried Fenugreek Leaves	**IC_50_**	Half-maximal inhibitory concentration
**AGE**	Agarose gel electrophoresis	**IKK**	I kappa B kinase
**Akt**	Protein kinase B (PKB)	**IL-1**	Interleukin-1
**ANOVA**	Analysis of Variance	**KATO-III**	Human gastric carcinoma
**ATCC**	American Type Culture Collection	**KDa**	Kilodalton
**Bax**	Bcl-2 associated X protein	**`LPS**	Lipopolysaccharide
**Bcl-2:**	B-cell lymphoma-2	**LSD**	Least significant difference
**Bcl-xl**	*B-cell lymphoma-extra large*	**MCF-7**	Michigan Cancer Foundation-7
**Bfl-1/A1**	Bcl-2-related protein A1	**MMP-9**	matrix metalloproteinase
**BFS**	Boiled Fenugreek Seeds	**MTT**	3-(4,5-dimethylthiazol-2-yl)-2,5-diphenyltetrazolium bromide
**c-MYC**	c-myelocytomatosis	**NEMO**	NF-κB Essential Modulator
**Cas-3**	Caspase-3	**NF-κB**	Nuclear Factor Kappa B
**cFLIP**	Cellular FLICE (FADD-like IL-1β-converting enzyme) Inhibitory Protein	**p53**	Protein 53
**Cox-2**	Cyclooxygenase-2	**P65 (RELA)**	REL-associated protein
**Ct**	Cycle threshold	**PCD**	Programmed Cell Death
**DMSO**	Dimethyl sulfoxide	**RT-PCR**	Real-Time Polymerase Chain Reaction
**ELISA**	Enzyme-linked Immunesorbent assay	**RPMI 1640**	Roswell Park Memorial Institute Medium
**FBS**	Fetal Bovine serum	**SAS 9.1**	Statistical Analysis Software
**GAPDH**	Glyceraldehyde-3-phosphate dehydrogenase	**SBs**	Strand breaks
**GFS**	Germinated Fenugreek Seeds	**SCGE**	Single-cell gel electrophoresis
**HCC**	Hepatocellular carcinoma	**SD**	Standard Deviation
**HCT-116**	Human Colorectal carcinoma	**Ser/Thr**	*Serine/Threonine*
**HepG2**	Hepatoma G2	**SFS**	Soaked Fenugreek Seeds
**HKgene:**	Housekeeping gene	**UFS**	Untreated Fenugreek Seeds
**HL-60**	Human leukemia cell line	**VERO**	*Verda Reno* (Green kidney)
